# Temporal alteration of serum bilirubin levels and its renoprotective effects in diabetic kidney disease: exploring the hormonal mechanisms

**DOI:** 10.3389/fendo.2024.1361840

**Published:** 2024-05-01

**Authors:** Can Cao, Shuwu Wei, Leijuan He, Chunyao Li, Yizhen Lu, Weiwei Sun, Yaoxian Wang

**Affiliations:** ^1^ Department of Nephrology and Endocrinology, Dongzhimen Hospital, Beijing University of Chinese Medicine, Beijing, China; ^2^ Department of Traditional Chinese Medicine, Dadushe Community Health Service Center, Beijing, China

**Keywords:** serum total bilirubin, diabetic kidney disease, hormonal mechanisms, eGFR slope, physiological range

## Abstract

**Objective:**

This current study represents a novel endeavor to scrutinize the correlation between the temporal alteration in serum total bilirubin (TBIL) concentrations and the rate of estimated glomerular filtration rate (eGFR). Additionally, this study aims to probe the plausible molecular mechanism underpinning the renoprotective effects of bilirubin concerning its hormonal characteristics.

**Materials and methods:**

In this study, a cohort of 103 patients diagnosed with DKD and receiving medical care at Dongzhimen Hospital were recruited and monitored over a period of 2-7 years. The progression of DKD was ascertained using a threshold of eGFR decline > -5.48%/year. To assess the relationship between the annual change in serum TBIL levels (%/year) and the slope of eGFR, multivariate binary logistic regression analysis was employed. Furthermore, the ROC curve analysis was employed to determine the cut-off value for TBIL levels (%/year).

**Results:**

The use of multivariate binary logistic regression models revealed that serum TBIL levels (%/year) exhibited a significant correlation with the slope of eGFR. Moreover, the ROC curve analysis indicated a cut-off value of -6.729%/year for TBIL levels (%/year) with a sensitivity of 0.75 and specificity of 0.603, in diagnosing eGFR decline >-5.48%/year.

**Conclusions:**

The findings of this study suggest that the sustained elevation of serum bilirubin concentration within the physiological range can effectively retard the progression of Diabetic Kidney Disease (DKD). Furthermore, the hormonal attributes of bilirubin may underlie its renoprotective effects.

## Introduction

Diabetic kidney disease (DKD) is a global health concern, accounting for the majority of end-stage renal disease cases (1). However, the rate of renal function decline varies among individuals (2), underscoring the need to identify factors that influence the progression of DKD. Previous studies have relied on a 30% reduction in estimated glomerular filtration rate (eGFR) as a surrogate endpoint for renal failure (3, 4). Unfortunately, this endpoint is not applicable to patients with high baseline eGFR or those experiencing a rapid decline in eGFR due to acute medication effects, thereby limiting its utility. The eGFR slope overcomes these limitations, thereby expanding the patient population that can be studied while significantly reducing the sample size and follow-up time (5). This metric provides an accurate reflection of disease progression in patients with varying stages of kidney disease. Therefore, this investigation aims to evaluate the effect of bilirubin on DKD progression by examining its influence on eGFR slope. In addition, our study used baseline TBIL levels and follow-up TBIL levels to calculate the annual change rate of bilirubin concentration on the basis of the follow-up time, which can better reveal the relationship between TBIL and DKD progression over time rather than the bilirubin concentration at a time point or simple numerical difference between the follow-up level and the baseline level.

Ahn et al. (6) observed an inverse relationship between serum TBIL levels and DN progression, independent of traditional risk factors. A retrospective longitudinal study demonstrated that individuals with the lowest range of serum TBIL levels had the highest cumulative incidence of CKD stage 3, indicating that serum bilirubin may serve as an early marker of CKD progression in T2DM patients (7). Eto et al. (8) further validated the clinical utility of serum TBIL (≤0.5 mg/dL) in predicting and identifying high risk of ESRD in DKD patients. These findings are rooted in the anti-inflammatory and anti-oxidative stress properties of bilirubin, which were examined in relation to serum bilirubin levels at a certain time point to delay DKD progression.

Currently, there is a gap in knowledge regarding the correlation between alterations in serum bilirubin levels and the progression of DKD. Recent research suggests that bilirubin possesses a novel function as a metabolic hormone that promotes gene transcription in nuclear receptors (9). In view of its hormonal properties, elevated levels of bilirubin within the physiological range could exert a positive influence on renal function. To date, no investigation has examined the association between annual change in serum TBIL levels and the eGFR slope, or the specific mechanisms that underlie its renoprotective properties. Hence, this study represents the first attempt to scrutinize these aspects of bilirubin’s influence on DKD progression.

## Materials and methods

### Subjects and data

The medical ethics committee of the Dongzhimen Hospital, Beijing University of Chinese Medicine, granted permission for the conduct of this retrospective study and the study conforms to the provisions of the Declaration of Helsinki. The data were obtained from the inpatient electronic medical records. The inclusion criteria were patients aged between 18 to 80 years, diagnosed with stage III~IV DN according to the Chinese guidelines for diagnosis and treatment of DN (10), and admitted to the Tongzhou Branch of Dongzhimen Hospital from January 2013 to December 2021. Exclusion criteria were patients with incomplete clinical information, history of kidney surgery, severe hepatic failure (levels of ALT, AST, and GGT exceeding three times the upper limit of normal range: ALT>120 U/L, AST>120 U/L, GGT>180 U/L), infection, malignant tumor, cirrhosis, acute complications, and acid-base disturbances within 1 month. Patients who were admitted to the nephrology department at least twice within the monitoring period of 2 to 7 years were considered eligible for the study.

### Laboratory assays

Blood samples were collected from the study participants after an overnight fast of at least 8 hours. The biochemical parameters including glycated hemoglobin type A1c (HbA1c, %), fasting plasma glucose (FPG, mmol/L), serum creatinine (Scr, µmol/L), blood urea nitrogen (BUN, mmol/L), uric acid (UA, µmol/L), alanine aminotransferase (ALT, U/L), aspartate aminotransferase (AST, U/L), glutamyl transpeptidase (GGT, U/L), total bilirubin (serum TBIL, µmol/L), direct bilirubin (DBIL, µmol/L), and indirect bilirubin (IBIL, µmol/L) were analyzed using an automatic biochemical analyzer (Beckman-DXC800, American). The eGFR was calculated using the CKD-EPI formula (11).

### Statistical analysis

Continuous variables that followed a normal distribution were presented as mean ± standard deviation (SD), and the independent samples t-test was utilized to compare the means between two groups. Skewed data (Kolmogorov–Smirnov test: p<0.1 for each) were presented as median (interquartile range). The Mann–Whitney U-test was used to compare the differences in the clinical characteristics. The chi-squared test (χ2 test) was applied to analyze categorical variables. Multivariate binary logistic regression was used to evaluate the relationship between the eGFR slope as the dependent variable and demographic, clinical, or laboratory variables as independent ones. The association between the eGFR slope and predictors was estimated using the Spearman correlation analysis. Data analysis was conducted using SPSS software (Statistical Package for the Social Sciences, version 25.0, Chicago). P < 0.05 was considered statistically significant.

The eGFR slope, as outlined by Greene, T (12) , defines the mean change in estimated glomerular filtration rate (eGFR) over time, usually calculated from the baseline measurement to a designated later time during the study’s follow-up period. This investigation defines the eGFR slope as the mean annual rate of eGFR change from baseline to specific time intervals across the follow-up span. DKD progression in this study is characterized by an eGFR slope exceeding -5.48% annually. The annual percentage change in eGFR and TBIL were calculated by dividing the absolute change in eGFR or serum TBIL by the baseline value, then by the follow-up time, and finally multiplying by 100. A cut-off value of -5.48% for the eGFR slope was established based on a previous reference (13). The study population was categorized into two groups based on their eGFR slopes: Group 1, with an eGFR slope>-5.48%/year, and Group 2, with an eGFR slope ≤ -5.48%/year.

## Results

### General data and correlation analysis

In this study, a total of 103 individuals with DKD were included. These individuals were classified into two groups based on their eGFR slope: group 1, comprising 40 patients with an eGFR slope >-5.48%/year, and group 2, comprising 63 patients with an eGFR slope ≤ -5.48%/year. At baseline, both groups were comparable in terms of their demographic characteristics, including age, gender, diabetes duration, monitoring time, SBP, DBP, and drinking habits, as well as all biochemical parameters. The detailed information is provided in **Table 1**.

**Table 1:** Main data on patients enrolled at the baseline of the study.

	Group1 Annual percentage in eGFR>-5.48%/year	Group2 Annual percentage in eGFR≤-5.48%/year	z/t/χ2 values	*P* value
Gender, males/females	25/15	43/20	0.361	0.548
Age, years	58.0 (16.5)	59.0 (15.5)	-1.016	0.310
Monitoring time, years	3.3 (1.3)	3.4 (1.4)	-0.802	0.422
Diabetes duration, years	12.2 ± 8.1	13.0 ± 7.02	-0.527	0.599
SBP, mmHg	133.1 ± 17.9	139.2 ± 20.9	-1.527	0.130
DBP, mmHg	79.5 (8.0)	80.0 (15.0)	-1.506	0.132
Drinking, yes (%)	10 (25)	16 (25.4)	0.002	0.964
CKD Stage,G1/G2/G3/G4	20/11/9/0	28/15/16/4	2.918	0.404
Scr, µmol/L	77.0 (31.7)	79.0 (54.0)	-1.046	0.296
eGFR, ml/min/1.73m^2^	89.9 (46.0)	84.4 (61.9)	-0.487	0.626
ACR,A2/A3	24/16	16/47	12.333	<0.001
ALT, U/L	19.5 (19.5)	17.0 (12.0)	-1.808	0.071
AST, U/L	19.0 (14.9)	18.0 (7.5)	-1.193	0.233
GGT, U/L	28.0 (26.5)	25.0 (20.3)	-0.423	0.672

DBP, diastolic blood pressure; SBP, systolic blood pressure.

ACR, A2:<300mg/g; A3: ≥300mg/g.

Group 1 had a median eGFR slope of 0.33%/year, ranging from – 5.45 to 26.6%/year, while group 2 had a median eGFR slope of -14.8%/year, ranging from −41.49 to −5.95. The median annual percentage change in serum TBIL was 0.38%/year, ranging from – 29.81 to 34.43%/year in group 1, and -8.25%/year, ranging from −13.81 to 22.81%/year in group 2. The annual percentage change in serum TBIL was significantly lower in group 2 than in group 1 (0.38 (11.3) vs -8.25 (14.7), z= -2.768, P= 0.006). The laboratory results showed that HbA1c, ACR, ALT, AST, serum TBIL, IBIL, and DBIL levels decreased significantly in group 2 after monitoring, while there was no significant change in these parameters in group 1. The detailed information is provided in **Table 2**.

**Table 2:** Results of laboratory analyses at the before and the after monitoring of the study for two groups formed according to the annual percentage change in eGFR.

	Group1 Annual percentage in eGFR>-5.48%/year			Group2 Annual percentage in eGFR≤-5.48%/year		
	Before	After	*P* value	Before	After	*P* value
Annual percentage change in eGFR,%/year		0.33(5.9)			-14.8(11.0)	
Annual percentage change in serum TBIL,%/year		0.38(11.3)			-8.25(14.7)	
eGFR, ml/min/1.73m^2^	89.9(46.0)	96.6(44.4)	0.460	84.4(61.9)	38.1(37.3)	<0.001
BUN, mmol/L	5.2(2.5)	6.4(3.1)	0.008	6.2(2.9)	10.4(9.0)	<0.001
Scr, µmol/L	77.0(31.7)	71.5(30.5)	0.039	79.0(54.0)	147.0(152.0)	<0.001
ACR,A2/A3	24/16	20/20	0.369	16/47	7/56	0.038
GLU, mmol/L	10.0(5.9)	10.2(5.7)	0.587	9.2(4.3)	7.7(4.3)	0.031
HbA1c, %	9.2(1.5)	8.7(3.0)	0.798	9.7(3.3)	8.2(2.5)	<0.001
ALT, U/L	19.5(19.5)	18.5(13)	0.231	17.0(12.0)	14.0(8.2)	<0.001
AST, U/L	19.0(14.9)	19.5(7.5)	0.214	18.0(7.5)	15.0(7.0)	<0.001
GGT, U/L	28.0(26.5)	26.5(20.5)	0.261	25.0(20.3)	24.0(11.8)	0.084
serum TBIL, µmol/L	12.6(7.8)	13.8(7.0)	0.941	12.2(7.6)	8.7(4.6)	<0.001
DBIL, µmol/L	3.4(2.1)	3.5(1.6)	0.411	2.8(1.9)	2.2(1.4)	0.010
IBIL, µmol/L	9.7(6.5)	9.7(6.4)	0.752	9.1(6.2)	6.4(2.9)	<0.001

ACR, A2:<300mg/g; A3: ≥300mg/g.

Given the potential association between several biochemical parameters (ACR, SCR, GLU, ALT, AST, serum TBIL, serum TBIL (%/year), IBIL, and DBIL) and the eGFR slope, we conducted a multivariate binary logistic regression analysis to identify potential protective factors for the annual percentage change in eGFR. Specifically, we included ACR, SCR, eGFR, DBIL, and serum TBIL (%/year) as covariates in our analysis. Our results, as presented in **Table 3**, indicate that DBIL was independently associated with a protective effect against changes in eGFR (OR 0.484, 95% CI 0.317-0.738, P=0.001), along with serum TBIL (%/year) (OR 0.919, 95% CI 0.875-0.965, P=0.001). In contrast, ACR was identified as an independent risk factor (OR 4.473, 95% CI 1.727-13.028, P=0.003), along with SCR and eGFR. The detailed information is provided in **Table 3**.

**Table 3:** Variables associated with the annual percentage change in eGFR(Binary logistic regression).

	Univariate analysis			Multivariate analysis		
	OR	95CI%	*P* value	OR	95CI%	*P* value
GLU, mmol/L	0.975	0.889~1.070	0.595			
ACR, A2/A3	4.339	1.897~10.201	0.001	4.743	1.727~13.028	0.003
HbA1c, %	1.129	0.908~1.404	0.275			
BUN	1.153	0.972~1.369	0.102			
SCR	1.010	0.998~1.023	0.090	1.056	1.020~1.094	0.002
GFR	0.997	0.984~1.009	0.583	1.053	1.014~1.093	0.007
ALB	0.989	0.907~1.078	0.802			
ALT, U/L	0.976	0.949~1.003	0.075			
AST, U/L	0.969	0.935~1.005	0.087			
GGT, U/L	0.996	0.988~1.006	0.448			
serum TBIL, µmol/L	0.924	0.857~0.996	0.038			
DBIL,µmol/L	0.680	0.508~0.909	0.009	0.484	0.317~0.738	0.001
IBIL, µmol/L	0.929	0.853~1.012	0.093			
serum TBIL,%/year	0.958	0.925~0.992	0.017	0.919	0.875~0.965	0.001
Gender	1.290	0.562~2.962	0.548			
Age	0.977	0.940~1.014	0.977			
Diabetes duration	1.015	0.962~1.071	0.595			
SBP	1.016	0.995~1.038	0.131			
DBP	1.025	0.983~1.070	0.243			
Drinking	1.021	0.410~2.545	0.964			

Spearman correlation analysis was performed to assess the association between eGFR(%/year) and various biochemical parameters including serum TBIL,%/year, DBIL, and ACR. The analysis revealed that serum TBIL,%/year and DBIL had a significantly positive correlation with eGFR(%/year) (rs value: 0.352, P <0.001; rs value: 0.228, P =0.021, respectively). Conversely, ACR showed a negative correlation with eGFR(%/year) (rs value: -0.338, P <0.001). These findings are presented in **Table 4**.

**Table 4:** Correlation of eGFR(%/year) and predictors.

Variable	rs values	P values
serum TBIL,%/year	0.352	<0.001
ACR	-0.338	<0.001
DBIL	0.228	0.021

Receiver operating characteristic (ROC) curves were utilized to analyze the endpoint of -5.48/year for the eGFR slope. Patients were divided into group 1 (marked as 0), with an eGFR slope greater than -5.48%/year, and group 2 (marked as 1), with an eGFR slope less than or equal to -5.48%/year. ROC curves were utilized to identify a cutoff value for serum TBIL,%/year that distinguished patients with a high risk of an annual percentage change in eGFR greater than -5.48%/year. The cutoff value with the highest accuracy was found to be -6.729% (sensitivity 75.0%; specificity 60.3%), and patients were subsequently categorized into two groups: Group 3, those with serum TBIL,%/year greater than -6.729%, and group 4, those with serum TBIL,%/year less than or equal to -6.729%.The detailed information of the two groupsis provided in **Table 5**.

**Table 5:** Results of laboratory analyses at the before and the after monitoring of the study between low or high serum TBIL.

	Group3 Annual percentage in serum TBIL>-6.729%/year (n= 55)			Group4 Annual percentage in serum TBIL≤-6.729%/year (n= 48)		
	Before	After	*P* value	Before	After	*P* value
Annual percentage change in eGFR,%/year		-5.6 ± 10.6			-14.4 ± 10.7	
ACR,A2/A3	26/29	18/37	0.119	14/34	9/39	0.232
Monitoring time, years		3.5 (1.4)			3.0 (1.3)	
eGFR, ml/min/1.73m^2^	90.34 (51.9)	77.6 (53.8)	<0.001	79.8 (63.0)	43.1 (20.9)	<0.001
BUN, mmol/L	5.5 (2.8)	7.2 (5.4)	<0.001	5.75 (2.7)	9.8 (6.6)	<0.001
Scr, µmol/L	77.0 (46.0)	86.0 (59.0)	0.003	79.0 (48.0)	134.5 (96.0)	<0.001
GLU, mmol/L	9.1 (4.1)	9.0 (4.7)	0.804	10.5 (6.8)	7.7 (5.4)	0.015
HbA1c, %	9.4 (3.2)	8.6 (2.3)	0.233	9.6 (3.1)	8.4 (2.8)	0.001
ALT, U/L	18.0 (17.1)	15.0 (13.1)	0.007	17.5 (14.0)	15.0 (10.3)	0.012
AST, U/L	17.0 (11.5)	17.0 (7.0)	0.014	19.0 (8.5)	17.0 (5.0)	0.010
GGT, U/L	24.0 (23.5)	23.0 (15.9)	0.047	27.0 (17.8)	25.5 (18.0)	0.346

**Figure 1:**
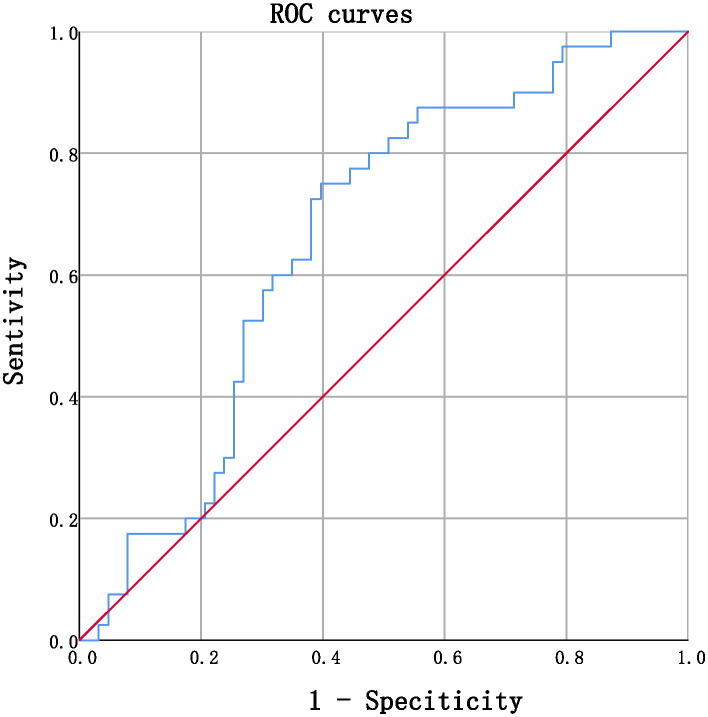
ROC curves in predicting annual percentage change in eGFR by serum TBIL (continuous, AUC= 0.662, *P*=0.006, CI% 0.557~0.768.

Upon comparing the laboratory results, it was observed that the eGFR after monitoring was significantly lower compared to before monitoring. However, in Group 4, a significant decrease was observed in the annual percentage change in eGFR as compared to Group 3, as evidenced by the mean values of -5.6 ± 10.6 and -14.4 ± 10.7, respectively (t= 4.149, P<0.001), as presented in **Figure 1**.

## Discussion

This investigation demonstrates a statistically significant association between annual change in serum TBIL levels within the physiological range and the eGFR slope, and highlights the independent protective effect of serum DBIL levels against DKD progression. These findings suggest that fluctuations in serum bilirubin concentration over time could be implicated in the progression of DKD, and that higher levels of bilirubin within the physiological range may have a beneficial effect in delaying DKD progression. Many previous studies have been tried to demonstrate the relationship between serum total bilirubin and DKD. Tafese R et al. (14) showed that the serum total bilirubin level was significantly lower in DN patients compared with non-DN patients and was significantly associated with an increased risk of DN patients. A 10-year observational cohort study in Japanese patients with diabetes was to evaluate the association of baseline serum TBIL levels with progression of DN and found that serum TBIL levels were negatively associated with PRD in diabetic nephropathy and its cut-off point was 0.5 mg/dL (8). Liu M et al. (15) showed that not only baseline TBIL, but also follow-up changes were significantly associated with DN incidence and progression based on a 5 years’ cohort study. Ahn KH et al. (6) conducted a retrospective observational longitudinal study of patients, and showed that serum bilirubin might be an early clinical marker for predicting the progression of CKD in patients with T2DM and preserved renal function. Our study used baseline TBIL levels and follow-up TBIL levels to calculate the annual change rate of bilirubin concentration on the basis of the follow-up time, which can better reveal the relationship between TBIL and DKD progression over time rather than the bilirubin concentration at a time point or simple numerical difference between the follow-up level and the baseline level. In addition, instead of analyzing the protective effect of bilirubin based on its anti-inflammatory and antioxidant properties, we tried to elucidate a completely new mechanism.

Our investigation reveals that patients with a faster annual decline in renal function exhibit a faster annual decline in serum TBIL levels. Specifically, patients with an eGFR slope of greater than -5.48%/year had higher serum TBIL (%/year) levels, while patients with an eGFR slope of less than or equal to -5.48%/year had lower serum TBIL (%/year) levels. Furthermore, our long-term follow-up analysis revealed that all bilirubin levels, including TBIL, DBIL, and IBIL, were significantly decreased in the group with a lower eGFR slope. Multivariate binary logistic regression models showed that serum TBIL (%/year) level was significantly associated with eGFR slope, while serum DBIL level was an independent protective factor for eGFR slope, and ACR was an independent risk factor. In addition, we found a significant positive correlation between eGFR (%/year) and serum TBIL (%/year) and DBIL, while ACR showed a significant negative correlation. These findings support the notion that annual change in serum TBIL levels may be associated with diabetic kidney disease progression. Higher levels of DBIL in the physiological range appear to be an independent protective factor for DKD progression, whereas ACR is an independent risk factor. Overall, our findings suggest that fluctuations in serum bilirubin concentration may indeed exert a renoprotective effects. Regular assessment of serum bilirubin levels and their variability may facilitate early prediction of DKD advancement. If a decline in serum bilirubin concentration is detected, interventions aimed at increasing bilirubin levels might be considered to slow down DKD progression. However, such interventions should be approached cautiously and require rigorous scientific research design to validate their efficacy and safety. It’s noteworthy that extremely high serum bilirubin levels are regarded as pathological and have been associated with increased mortality (16). while fluctuations in serum bilirubin concentration hold promise as a potential protective factor against DKD progression, further scientific research is warranted to substantiate these findings and establish the feasibility of clinical interventions aimed at modulating bilirubin levels to mitigate DKD progression.

The pathogenesis of DKD mainly involves damage to the glomerulus, with podocyte injury being a key hallmark of this condition. Podocyte injury manifests as a disappearance and fusion of podocyte foot processes, leading to the accumulation of damage over time and ultimately resulting in podocyte apoptosis and shedding, which results in a substantial increase in proteinuria. This process is observed in the early stages of DN, and is a major driver of disease progression. Proression proteinuria is one of the most important prognostic risk factors for DN, which promotes the development of DN to ESRD (17). Among the various morphological features, the decreased number of glomerular podocytes was the strongest predictor of DN progression, with fewer podocytes indicating the faster the progression of DN. Dyslipidemia is an independent risk factor for the development of DN (18). High glucose can induce lipid droplet deposition, enabling the continuous accumulation of the extracellular matrix and progressive fibrosis in the kidneys with the progression of DN (19, 20). Previous studies have initially shown the underlying molecular mechanisms of this developmental process: High glucose causes intracellular lipid accumulation and phenotype changes in the podocytes, which can cause the podocytes to lose their various morphological structure and function, and damage the integrity of glomerular filtration barrier and the synthesis of extracellular matrix components such as collagen, thus producing proteinuria and promoting DN progression (21). Herman et al. (22) reported severe lipid deposition and increased intracellular lipid droplets in renal biopsies of DN patients, while the levels of several genes involved in the fatty acid oxidation pathway were significantly downregulated, including PPAR α. Activated PPAR α significantly reduced triglyceride levels, and moderately reduced total cholesterol levels and low-density lipoprotein cholesterol levels, thus increasing high-density lipoprotein cholesterol levels (23). PPAR α is expressed in kidney podocytes, and activated PPAR α is able to reduce albuminuria and improve insulin resistance (24). Activation of PPAR α may be a favorable factor for DN progression. Recent studies suggest that bilirubin has a completely new function as a metabolic hormone for transcription of the genes that drive the nuclear receptors (9). Higher levels of bilirubin in the physiological range may have beneficial effects on the kidney due to this hormonal characteristics. Bilirubin can bind directly to PPARα and increase transcriptional activity. Stec et al. (25) showed that global PPAR α knockout mice have a reduced genetic response to bilirubin treatment, particularly hepatic fibroblast growth factor-21, a well-characterized PPAR α target gene. The hormone must bind directly to the target receptor to exert its effects. Although bilirubin may have an unknown role in activating other pathways, PPAR α has been shown to be a direct binding protein for bilirubin as a ligand agonist. Recent studies indicated that bilirubin only selectively bound PPAR α and did not interact with PPAR γ or PPAR δ (26). It is therefore speculated that the specific mechanism by which higher levels of bilirubin delay DN progression is related to the hormonal properties of bilirubin. Bilirubin flows through the blood over time and gradually enters the cell through the uptake system, continuously binding to the target receptor PPARα within the cell, resulting in core pressurized protein translated into coactivators to activate transcriptional control of the genes, which can further increase the downstream fat burning, improve insulin resistance and lipid metabolism disorders, regulate blood glucose levels, and thus play a role in delaying the progression of DN. Although the results obtained in this clinical study cannot directly reveal the intrinsic mechanism by which bilirubin as a hormone exerts a protective effect on DN, they showed that with the gradual increase of total bilirubin concentration in the physiological range over time, it may delay the progression of DN, which has a positive effect on the above mechanisms.

ROC curves were utilized to determine the optimal cutoff value for serum TBIL (%/year) that would identify patients at high risk of annual percentage in eGFR > -5.48%/year. The cutoff value that provided the highest accuracy was found to be -6.729%, with a sensitivity of 75.0% and specificity of 60.3%. Using this threshold, patients were divided into two groups: Group 3 comprised individuals with serum TBIL (%/year) > -6.729%, while group 4 included those with serum TBIL (%/year) ≤ -6.729%. Comparative analysis of laboratory data demonstrated that the eGFR after monitoring was significantly lower than that prior to monitoring, with the eGFR slope in group 4 being considerably lower than that observed in group 3. Furthermore, the results indicated that the serum creatinine and serum urea nitrogen levels were significantly elevated, reaching pathological levels in group 4, thereby validating the association between a more rapid decrease in TBIL levels over time and a more pronounced decline in eGFR slope, as well as greater impairment of renal function. Notably, our retrospective investigation relied solely on the clinical data of the participants, without any molecular information. Therefore, it remains necessary to verify the precise molecular mechanism by which bilirubin operates as a hormone and confers protection to renal function. Owing to the limitation in sample size, the current study acknowledges the potential impact on result stability and reliability. Therefore, it is recommended to undertake future investigations with a larger scale, encompassing multiple centers and employing a prospective methodology to enhance the robustness of the findings. The augmentation of serum bilirubin concentration within the physiological range over an extended period is advantageous in retarding the progression of DKD, whereby the hormonal capacity of bilirubin might serve as a prospective molecular mechanism underlying its renoprotective effect. These findings could open up novel avenues for clinical intervention and targets to delay the progression of DKD.

## Data availability statement

The raw data supporting the conclusions of this article will be made available by the authors, without undue reservation.

## Ethics statement

This study was approved by Ethics Committee of Dongzhimen Hospital Affiliated to Beijing University of Chinese Medicine (registration code:2023DZMEC-134). All experiments were performed in accordance with the ethical standards laid down in the 1964 Declaration of Helsinki and its later amendments as revised in 2008. The ethics committee board waived the requirement of written informed consent for participation from the participants or the participants’ legal guardians because since the study involved the utilization of data collected from inpatient electronic records.

## Author contributions

CC: Data curation, Formal analysis, Methodology, Writing – original draft. SW: Writing – original draft, Data curation. LH: Data curation, Writing – original draft. CL: Investigation, Writing – original draft. YL: Investigation, Writing – original draft. WS: Project administration, Writing – review & editing. YW: Project administration, Writing – review & editing.
